# Global Health Actors Claim To Support Health System Strengthening—Is This Reality or Rhetoric?

**DOI:** 10.1371/journal.pmed.1000059

**Published:** 2009-04-28

**Authors:** Bruno Marchal, Anna Cavalli, Guy Kegels

**Affiliations:** Department of Public Health, Institute of Tropical Medicine–Antwerp, Antwerp, Belgium

## Abstract

Bruno Marchal and colleagues argue that most current strategies aimed at health systems strengthening remain selectively targeted at specific diseases.

**Summary Points**

- Health system strengthening (HSS), the new buzzword in discussions about international health, is in danger of becoming a container concept that is used to label very different interventions.
- Many global health initiatives and agencies (which we term “global health actors”) claim that their activities support HSS.
- Most current HSS strategies are in fact selective, disease-specific interventions, and their effects may undermine progress towards the long-term goal of an effective, high-quality, inclusive health system.
- To make use of the window of opportunity for redefining HSS, a number of obstacles must be overcome. These include defining the exact objective of HSS strategies and finding the right balance between a health system's role in disease prevention versus treatment.

## “Health System Strengthening”: A New Buzzword

The recent explosion in the number of global health initiatives has dramatically changed the landscape of public health and international aid. During an initial honeymoon period, these initiatives started implementing their disease-specific activities in low-income countries. But the honeymoon is over, and there is now an increasing realisation that such initiatives ignore a wider problem—existing health systems in the developing world are fragile and unable to provide effective health services, especially in sub-Saharan Africa [1],[2]. Simultaneously, there is a growing consensus that effective global initiatives require well-functioning health systems [3]. As a result, health system strengthening (HSS) in low-income settings is now regarded, in the words of Alaka Singh at the World Health Organization (WHO), as “the ‘first-order’, immediate/medium-term goal to create the necessary enabling institutional and systemic environment to achieve and sustain ‘higher order’ MDGs [Millennium Development Goals] in the long(er) run” [4].

Despite this new attention upon HSS, the term remains a vague concept, with varying definitions and strategies for HSS, and varying ideas about the role attributed to the health system in improving public health. In this Policy Forum, we argue that most current HSS strategies are selective (i.e., they target a specific disease), and their effects may undermine progress towards the long-term goal of effective, high-quality, and inclusive health systems. There are, however, signs that the main actors in global health are aware of these risks, and a new window of opportunity for redefining HSS may be emerging. In order not to miss this opportunity, we urgently need a systemic approach to HSS that is contextual and that fits the countries' agendas first. Focusing on country health systems with limited resources, we aim to stimulate the debate on HSS and to suggest a way forward.

## Methods

The terms “global health initiative” and “global health partnership” are much used but ill defined. They cover very different groups of actors [5]. In this paper, we discuss international initiatives and key agencies working in the domain of HSS. Acknowledging their different nature, we use in this paper the term global health actor (GHA) for reasons of simplicity.

In order to document the views, definitions, and strategies of GHAs, we initiated our literature review by searching GHA Web sites for key strategic documents. The aim of the review was not to be inclusive, but to uncover key examples from the wide range of actors and HSS interventions. We then searched PubMed and Google Scholar using key words including “health system strengthening”, “global health initiatives”, and “vertical programmes”. Through repeated snowballing, other documents were identified, including grey literature. We used a form of concept mapping [6] (see also http://en.wikipedia.org/wiki/Concept_map) to identify key constructs used by GHAs and compared these constructs with their actual health interventions. A more detailed section on methodology is presented in Text S1.

## Our Key Findings

### Current HSS Programmes and Activities

Our review found that, on the ground, very different interventions are implemented by GHAs. These can be categorised as (1) providing inputs or resources, (2) reinforcing capacities of health services that are directly related to implementation of disease-control programmes, and (3) integrating programme activities into general health services. (See Text S2 for a detailed description.)

First, the provision of inputs and resources by GHAs is often presented as “health system support”. Such provision consists of inputs of material resources (infrastructure/rehabilitation, equipment, transport, communication) or financial resources. It may be targeted at a specific disease and supplementary to existing (governmental) funding for that disease. For instance, the GAVI Alliance (http://www.gavialliance.org/) supplements governmental funding for childhood immunisation [7]. It can also be put in a single “basket” of funds at national or local level that contributes to the national plan (e.g., funding of baskets by the Global Fund to Fight AIDS, Tuberculosis and Malaria [The Global Fund; http://www.theglobalfund.org/] in some countries).

Second, reinforcing programme-linked capacities targets health system functions that are essential for implementation of GHAs' programmes. Preferred means include technical assistance (often from expatriates) and training. The Global Network for Neglected Tropical Diseases (http://globalnetwork.org/), for instance, strengthens institutional capacities to plan, implement, and monitor the control, elimination, or eradication of neglected diseases [8].

Third, some GHAs, such as the Joint United Nations Programme on HIV/AIDS (UNAIDS; http://www.unaids.org/) [9] and Roll Back Malaria (http://www.rollbackmalaria.org/) favour integration of programme activities into general services. Roll Back Malaria's strategy includes strengthening drug procurement and distribution, quality control of laboratories, training, and monitoring of drug quality [10]. The African Programme for Onchocerciasis Control (http://www.who.int/apoc/en/index.html) presents the integration of community-directed ivermectin distribution into the existing health services as a means to strengthen the health system [11].

WHO and the Global Fund propose a “diagonal approach” to HSS (i.e., disease-specific outcomes are achieved by improving health systems). The starting point for such an approach is identifying which aspects of the health system are getting in the way of achieving outcomes related to malaria, tuberculosis (TB), or HIV/AIDS. This should then inform the design of a specific strategy to address the bottlenecks in such a way that both specific health outcomes and system-wide effects are achieved. Successful examples of such a diagonal approach, however, have not yet been documented [12].

### A Selective Approach to HSS

We found a clear gap between the language used by GHAs and their actual activities. Virtually all GHAs claim to support health systems, but instead they focus on disease-specific interventions or on activities targeting system functions essential for implementation of their own programmes. “Rapid-impact interventions” and measurable short-term outcomes are emphasised. In practice, micro-level solutions (incentives and support for general health services carrying out programme activities) or support to specific sub-systems essential for effectively implementing the programme (drug delivery, surveillance, etc.) are the norm.

GHAs identify weak health systems as the major barrier to the success of their programmes, but their responses tend to focus on their own specific objectives. Their HSS strategies are essentially a means to deliver targeted interventions more efficiently, rather than being strategic and directed towards the root causes of health system weaknesses. Therefore, HSS efforts of most actors can be more accurately described as selective HSS interventions.

### Definitions and Justification of HSS

In order to try and understand why GHAs choose such selective strategies, we briefly describe GHAs' policies on HSS. (More details are provided in Text S2.) WHO remains potentially the most important actor in this debate. Its World Health Report 2000 defines health systems “to include all the activities whose primary purpose is to promote, restore or maintain health” [13]. Its framework of health systems and performance is based on the four key functions of stewardship, resource mobilisation, service provision, and financing. In 2006, WHO circulated a revised framework on health systems. This conceives the health system as made up of six building blocks: policy, financing, human resources, supply system, service management, information, and monitoring systems. HSS is defined as “building capacity in critical components of health systems to achieve more equitable and sustained improvements across health services and health outcomes” [14].

The GAVI Alliance's definition of “health system” rephrases the World Health Report 2000 definition to fit immunisation programme functions [15]. GAVI recognises that immunisation coverage is often constrained by general health system barriers [5]. Its interventions tend to focus on strengthening those functions that are essential for good implementation of immunisation programmes [16]. As we will see below, GAVI's HSS window now offers opportunities to strengthen capacities not directly related to immunisation.

Although the Global Fund has funded HSS in a variety of ways, its opinions on health system support remain divided. Gradually, health workforce, management capacity, and governance have been given more attention, as well as the system-wide effects of the interventions it funds [12]. The Global Fund now proposes a “diagonal approach”, in which health system constraints to achieving outcomes related to malaria, TB, or HIV/AIDS are targeted by interventions that strive for specific health outcomes and positive system-wide effects [17]. In practice, the Global Fund calls for proposals addressing health system weaknesses through a “cross-disease approach” that should benefit more than one of the three diseases (“cross-cutting”) [18].

The HSS policies of most other GHAs are far less well developed. For instance, The US President's Emergency Plan for AIDS Relief (PEPFAR; http://www.pepfar.gov/) mentioned the term “health system” sparingly in its 2004 strategy paper [19]. In practice, it adopts a bilateral programme approach in its partnerships that mostly bypasses existing public institutions. More recently, it stated that capacity building within the public system is an objective, focusing on task shifting, training, and retaining health workers and building networks to support health workers [20]. Roll Back Malaria proposes to deliver malaria interventions through integrated health systems, and in doing so, to strengthen their capacity to deliver care for other diseases [10]. UNAIDS does not explicitly define HSS, but its general strategy includes building on the existing health infrastructure, increasing the number and skills of health workers, and coordinating and integrating services [21]. The US Agency for International Development (http://www.usaid.gov/) provides support to HSS through its “Global Health Systems Programs”, which focus on “priority” services in the domain of maternal and child health, including commodities, health care financing, health information, health workers, and policy reform [22].

In short, GHAs provide various reasons to justify HSS, ranging from being a means to reach programme-specific objectives or to scale up interventions, to a means for consolidating results and ensuring sustainability. Unsurprisingly, most agencies mention the Millennium Development Goals. Although all major agencies state that health system strengthening is important, most are unclear about their definition of a health system. Similarly, different definitions of HSS are circulating. Some use the definition presented in the WHO report commissioned by the Global Fund in 2006, while others present their own description (e.g., three GHAs use their own definition—the US Agency for International Development, the Stop TB Partnership [http://www.stoptb.org/], and Roll Back Malaria). Given this huge variety, we need a definition of HSS that is both shared and consistently applied by all actors.

## Analysis: From a Comprehensive Discourse to a Selective Practice

The selective, disease-specific nature of most current HSS strategies should not surprise us. True to their narrow focus, GHAs favour vertical programmes, which they consider the most efficient method to implement their activities. Furthermore, some actors appear to perceive health systems as “bottomless pits” in which external support disappears without a trace [23]. Instead of investing in long-term strengthening of national stewardship capacity, they prefer lifting specific health system constraints that impede progress towards their objectives.

Two problems arise. First, the vague definition of HSS allows GHAs to stick the label “health system strengthening” on any health-related capacity strengthening activity. Such liberal use of the word, for reasons of political correctness, turns “HSS” into a meaningless container concept.

Second, there are still doubts regarding the effectiveness of many global health initiatives [24]–[26]. No less important is the real risk of undermining existing services given the enormous financial leverage of some GHAs. In Uganda, for example, the total Ministry of Health budget for 2005 (US$112 million) was eclipsed by funding for AIDS from PEPFAR, the Global Fund, and the World Bank's Multi-Country HIV/AIDS Program (US$167 million). A similar situation occurred in Ethiopia. In both countries, the ministry had to outsource key management functions for these programmes due to inadequate capacity at national level [27].

Most GHAs now realise that their activities may also have negative effects (“system-wide effects”). These can be categorised as “duplication”, “imbalances”, and “interruptions” (adapted from Phyllida Travis and colleagues' framework [28]). *Duplication* is defined as multiplying efforts by developing parallel, non-integrated systems. The Multi-Country HIV/AIDS Program and PEPFAR are prime examples of agencies setting up parallel planning, operations, and monitoring systems [29]. Parallel systems undermine local decision-making autonomy and lead to inefficiency [30]. Duplication also includes setting up parallel delivery systems, for instance operated by non-governmental organisations. *Imbalances* are defined as the creation of differences in resource allocation and utilisation within the health workforce. Often, GHAs draw personnel out of general health services into their programmes [5],[15],[31]. These risks are not imagined: in Nepal, for example, health workers preferred to work with National Immunisation Day programmes because of the higher per diem rates [32]. We define *interruptions* as displacement of routine services due to programme activities such as training, fieldwork, administration, and accounting. In Cambodia, campaigns on HIV/AIDS, malaria, TB, and birth spacing led to reduced coverage rates of the routine immunisation programme [33].

## Discussion

### Clearing Some Hurdles

There are some promising signs to suggest that some GHAs recognise the need to redefine their approach to HHS. For example, in 2005, the GAVI Alliance created a new funding “window” for HHS support, to assist countries to increase their immunisation coverage in a way that strengthens their health systems. The alliance recommended that applicants should focus on the health workforce, management at the district level, and supply and maintenance systems. Although HSS interventions funded by GAVI should still improve immunisation coverage [16], presentations at the Geneva Health Forum 2008 on Liberia [34] and Ethiopia [35] show how GAVI funding is being used outside the narrow immunisation domain.

In order to make the best of these types of windows of opportunity to redefine HSS, we need to tackle a number of obstacles. The first is addressing the question of what the aims of HSS should be. To answer this, one needs to clarify the goals that a health system should pursue. Currently, most HSS actors do not explicitly address this question. The HSS framework proposed by WHO describes the goals as: “to achieve more equitable and sustained improvements across health services and health outcomes” [13]. Refining such a mission statement could be a starting point to better align GHAs and national actors; the current HHS framework is rather generic and open to widely diverging interpretations.

Second, some deep-seated tensions must be addressed. These result from structural divisions between the public and private sector (including both for-profit and not-for-profit providers), and between formal and informal providers, which affect funding, workforce deployment, and especially regulation. Other tensions arise from the conflicting vertical and horizontal approaches to health service organisation. Typically, disease programmes narrowly aim at controlling or eliminating a specific health problem. In contrast, “horizontalists” would strive to ensure provision of care that responds to the overall needs of the population. However, to frame the HSS discussion in terms of the old vertical–horizontal conflict is counter-productive, since both approaches are needed. Disease programmes contribute to the health system's role in public health protection. Immunisation campaigns, for instance, aim at primary prevention, while regular population screening for trypanosomiasis also includes secondary prevention (and allows for case treatment). Nevertheless, most common health problems require comprehensive services to ensure accessible and high-quality care to those who need it. This is the responsive role of a health system—examples include orthopaedic services for road accident victims or out-patient clinics for patients with acute or chronic diseases. The true question is how to balance these roles, and subsequently balance the funding and provision of the right mix of services.

A third obstacle to effective HSS is the complex nature of health systems. Both for analysing and implementing HSS, health services should be considered as open systems. Health services cannot operate in a vacuum: they draw resources from their environment and need to be responsive to their users. Health services are also complex adaptive systems as opposed to mechanical systems [36]. A complex adaptive system is defined “as a collection of individual agents with the freedom to act in ways that are not always predictable, and whose actions are interconnected so that one agent's actions change the context for other agents” [37]. Current health systems are made up of numerous actors, including public-oriented and private providers, formal and informal providers, professional, non-professional, and lay providers, and conventional/Western and traditional providers. The relationships between population and users, providers, health authorities, and governing bodies are quite dynamic. Contributing to the complexity, many approaches to health service organisation co-exist: family medicine, vertical programmes, social interventions, educational programmes, hospital services, integrated networks of providers, etc. As a result of this complexity, it is often difficult to accurately identify weaknesses of a health system.

- Weaknesses at one level may be the consequence of a root cause at another level. For example, at first sight, low immunisation coverage may be due to insufficient numbers of health workers in rural areas. However, inadequate staffing may be due to the fact that rural health facilities are unattractive places to work, with poor working conditions, poor staff housing, and inadequate supervision. All of these local factors may in turn have root causes at district or national level, and often at both. Because of the linkages between the different actors and levels of a health system, an effective HSS approach is based on the analysis of root causes of weak performance at the various interlinked levels of health system: community, operational services (first and second line, the district level, control programmes) and national level and international actors.
- Given that health services operate as open systems, one must understand their context. Ethiopia's strategy of deploying “health extension workers” to provide basic curative and preventive health services in every rural community may bring health care closer to the population. But such a strategy would not necessarily work in the same way in South Africa given major differences in health workforce, staffing levels, rural communication infrastructure, etc. Solutions effective in one setting are not necessarily so in another.
- A critical analysis of what we know about capacity building found that capacity building is essentially a dynamic, continuous, and long-term process [38]. It virtually always includes major personal, organisational, and institutional change, which means that there are no quick fix solutions. Translated to the HSS debate, GHAs and other actors should adopt contextualised approaches and a long-term perspective in funding and supporting national actors.

Fourth, to effectively strengthen health systems, the health workforce is of key importance. In a number of countries, internal and external brain drain of staff is compounding acute and chronic imbalances of health workers [39]. To make matters more complicated, some GHA interventions may contribute to these imbalances by draining staff from regular health services into their programmes [5],[31].

### The Way Forward

In the first place, GHAs and country health authorities should analyse their goals and see how their policies and programmes contribute to ensuring both protective and responsive health system functions. Donor coordination is now firmly on the agenda—the International Health Partnership (http://www.internationalhealthpartnership.net/), for example, has adopted an inter-agency coordinated process and common work plan for working towards the Millennium Development Goals [40]. However, in many countries, more care could be taken to align global health initiatives with national priorities.

Second, funding for health should be restructured to avoid displacement of aid to the detriment of support for health systems, which has probably already occurred [41]. Many debates in international health and development focus on the feasibility of intervention packages in terms of cost-effectiveness and sustainability. However, instead of adapting interventions to current budget ceilings, the latter also need review. In 2000, in Abuja, Nigeria, African Heads of State committed to allocating at least 15% of their annual budget to improving the health sector, but for the poorest African countries, this goal may be too ambitious. Other mechanisms to ensure long-term, predictable funding are required [42]. In such cases, sustainable financing is more important than local financial sustainability. Gorik Ooms and colleagues propose that the Global Fund should be retooled to this effect [43].

Third, increased and sustained health financing requires adequate absorption and implementation capacities at central and operational level. GHAs should avoid undermining existing local capacity to manage and organise responsive health services. A key example is the health workforce. Developing and adhering to a code of recruitment could reduce poaching of personnel from existing services. Better yet, GHAs could contribute to investment in training and in raising salary and wage levels, not only for “their” programme staff, but for the whole workforce, while countries would need to lift bureaucratic barriers to effective human resources management. Kenya's Ministry of Health is reported to require 18 months to fill vacancies in the face of thousands of unemployed nurses [44].

Finally, research priorities include, first, the development of analytical frameworks that allow health service managers and national policy makers to identify negative and positive effects of GHAs and to develop appropriate strategies. Second, while evaluations should assess effectiveness of HSS interventions, we also need to better understand “what works, how, for whom and in which context” [45]. In other words, we need to open the black box between the intervention and its outcomes. Realistic synthesis is an approach to evaluation that provides a systematic framework to do this by identifying how interventions work out in practice and which context conditions are essential for success [46]. In a second wave, systematic assessment of interventions could lead to developing typologies of countries, health policies, and HSS interventions and provide policy makers with context-specific insights to guide their choice of interventions.

## Conclusion

The renewed attention upon health systems is welcome, but many GHAs are doing no more than putting old wine in new bottles. They claim that their selective practices are contributing to strengthening systems, while in reality the opposite might be the case.

A consensus on the exact objective of HSS strategies would be a first step forward. HSS should contribute not only to the protective function of a health system, but also to its responsive function. Second, a number of analytical principles can be used to deal with complexity in the design of HSS interventions. Third, an increase in funding for health systems will not be enough unless it is sustained and well balanced between the two main goals of health systems (prevention and treatment). Finally, robust methods to learn from current interventions are urgently needed.

## Supporting Information

Text S1 Detailed methodology.
(44 KB DOC)

Text S2 A summary of policies and interventions of global health actors in the domain of health system strengthening.
(60 KB DOC)

## Abbreviations

GHA: global health actor
HSS: health system strengthening
PEPFAR: President's Emergency Plan for AIDS Relief
TB: tuberculosis
WHO: World Health Organization
