# Supplementary material for: Global Health Actors Claim To Support Health System Strengthening—Is This Reality or Rhetoric?
Source: PLoS Med. 2009 Apr 28;6(4):e1000059. doi: 10.1371/journal.pmed.1000059 (PMC2667637; doi:10.1371/journal.pmed.1000059)
Supplement: Text S1 — Detailed methodology. (44 KB DOC) [file pmed.1000059.s001.doc]

### Supporting information file 2

# A summary of policies and interventions of global health actors in the domain of health system strengthening

Reviewing the literature, one is struck by a bewildering variety of definitions of the term ‘health system strengthening’. In this section, we briefly present the constructs and concepts of some of the major global health actors (GHA).

In its World Health Report 2000, WHO proposes a framework of health systems and performance based on 4 key functions: stewardship, resource mobilisation, service provision and financing [1]. Later, WHO convened the Task Force on Health Systems Research to suggest topics for international research to generate the knowledge necessary to improve health systems and, thus, the prospects for attaining the MDGs [2]. In 2006, the Global Fund commissioned a WHO review of the opportunities for GHA to strengthen health systems. WHO proposed an action framework that conceived the health system as made up by 6 critical components [3]. WHO considers that health services, health workforce, health information system, provision of drugs, health financing and leadership & governance are the key building blocks [4]. Health system strengthening is then defined as “*building capacity in critical components of health systems to achieve more equitable and sustained improvements across health services and health outcomes*” [3]. WHO considers HSS as a strategy for countries to achieve national health goals, a necessary contribution to reaching global and regional development goals, including MDGs, and finally a means to consolidate early results [3].

The GAVI Alliance’s definition of ‘health system’ is based on the World Health Report 2000 framework on *health systems* (4 key functions) [5] and the six building blocks [6], but it rephrases them in a constraining manner to fit the immunization programme functions. The justification for GAVI to work on HSS is the recognition that immunization coverage is often constrained by health system barriers that are not specific to immunization. Their interventions therefore tend to focus on strengthening those functions that are essential for good implementation of immunisation programmes. HSS is therefore instrumental in reaching its own health problem-specific objectives and to improve immunisation-linked outcomes [7,8,9].

Spending more than 50% of its funds on HSS, the Global Fund is a major player. The Global Fund shares the WHO health system definition of the six building blocks [3] and acknowledges that “*it is impossible to scale up services to any significant extent without a stronger system*”. It calls for a “diagonal approach”, where the starting point is the identification of health system constraints to achieving outcomes related to malaria, TB or HIV/AIDS. On the basis of these constraints, a specific strategy is developed to ensure that both specific health outcomes and positive system-wide effects are achieved. The programmatic interventions it funds are therefore assumed not only to have a direct impact on the targeted diseases, but also on the functioning of the broader health care system. As such, the Global Fund considers that the programmes it funds will affect the policy environment, the public/private mix, the human resources and pharmaceuticals and commodities [10][[1]](#footnote-2). However, within the Global Fund, opinions about modalities of health system support remain divided, particularly concerning the need of focusing on HSS as a disease specific effort or to tackle it through an additional HSS component [11].

The World Bank’s new Health Nutrition and Population (HNP) strategy focuses on health system strengthening, health financing, government leadership and international community programmes. HSS is described as “*putting together the right chain of events”* consisting of financing, insurance, logistics, provider payment and incentive mechanisms, information, well-trained personnel, basic infrastructure and supplies [12]. It considers health financing, insurance, demand-side interventions, regulation and systemic arrangements for fiduciary and financial management as its comparative advantage in the HSS domain. In practice, the strategy recognizes that instituting the right incentives for efficiency will be insufficient in low income countries and that to effectively reinforce the health system’s delivery capacity, the supply-side, fiscal spaces and international financing need to be tackled as well.

Strengthening the capacity of health care programmes in low- and middle-income countries to deliver the comprehensive services needed by people living with HIV has always been a focus of UNAIDS and its partners [13]. This agency does not explicitly define HSS related to HIV control activities, but its general strategy includes building on and strengthening the existing health infrastructure, increasing the number and skills of health care workers, and coordinating and integrating services. HSS is then instrumental in scaling up interventions for prevention, care and support of PLWHA ([14,15].

In its 2004 strategy paper, PEPFAR uses the term ‘health system’ sparingly and only in relation to building capacities to reduce stigmatization, to the provision of drugs and supplies and to research [16]. For PEPFAR, the justification of focusing on activities of lasting benefit is to ensure a sustainable response to the AIDS pandemic [17]. In practice, PEPFAR adopts a bilateral programme-approach in its partnerships that often bypasses existing structures. More recently, PEPFAR stated that capacity building within the public system is a major objective, whereby it focuses on task shifting, training and retaining health workers and building health networks to support health workers [17].[[2]](#footnote-3) The evaluation of the progress by the Institute of Medicine [18] stresses the need to focus on long-term sustainability by investing more in the health workforce, but does not mention openly health systems.

Through its ‘Global Health Systems Programmes’, USAID provides support to health systems strengthening in different countries as *“an integral part of creating effective basic services in maternal and child health and other priority services*”. HSS is defined as *“a continuous process of implementing changes in policies and management arrangements within the health sector”*. The priority technical areas include commodities (pharmaceutical procurement and management), health care financing, health information, health workers and policy reform. The participation in the area of health systems is considered as necessary to create *“effective basic services in maternal and child health and other priority services” [19]*.

The Stop TB Strategy recognizes health system weaknesses in coverage, quality and human resources as a bottleneck for the TB program, and considers HSS as a key component of the strategy. HSS is considered in a functional approach, allowing the access to good quality of TB care [20]. The Stop TB strategy calls for national programmes to engage in HSS by paying attention to policies, human resources, management, funding, service delivery and information. Innovative implementation strategies should contribute to HSS [7], but more specific interventions are not proposed, except the PPM DOTS strategy. While strongly promoting private sector involvement in TB control, PPM DOTS (Public Private Mix Directly Supervised Treatment Short course) states that it will “*contribute to health system strengthening, actively participate in efforts to improve system-wide policy, human resources, financing, management, service delivery, and information systems*” [3].

Roll Back Malaria proposes to deliver malaria interventions through integrated health systems, and in doing so, strengthen their capacity to deliver care for other diseases as well [21].

The Neglected Diseases Network considers HSS as a strategic area to combat Neglected Tropical Diseases (NTD) [22], but does not present a definition of ‘health system’. Existing health systems are considered as the optimal setting to deliver control strategies for NTD and therefore their capacity for diagnosis, treatment, case management and surveillance needs to be strengthened. HSS is therefore considered as instrumental for sustainable NTD control. After 30 years of *ivermectin* community-directed mass treatment programmes, the African Programme for Onchocerciasis Control (APOC) is advocating for HSS. It proposes this as a strategy to ensure sustainability of onchocerciasis control once the regional programme will stop its support to the countries [23].

For the Disease Control Priorities Project, well-performing health systems are critical to effectiveness, efficiency and equity of disease control programmes, and to this end, they need to be strengthened in key domains. Health systems consist of different dimensions: stewardship and regulation, organisational structures and financing, human resources, quality assessment and assurance, and resources for efficient use at system- and service-level [24]. These authors suggest that in low-capacity environments, vertical programmes should preferably be used. In function of general development of the system, a shift to less-selective and more horizontal approaches in financing and service delivery is made.

### References

1. WHO (2000) The World Health Report 2000 - Health systems: improving performance. Geneva: World Health Organisation.

2. Task Force on Health System Research (2004) Informed choices for attaining the Millennium Development Goals: towards an international cooperative agenda for health-systems research. Lancet 364: 997-1003.

3. WHO (2006) Opportunities for global health initiatives in the health system action agenda, Working paper n° 4. Geneva: World Health Organisation.

4. WHO (2007) Everybody's business: Strengthening health systems to improve health outcomes: WHO's framework for action. Geneva: World Health Association.

5. GAVI (2001) Strategic Framework for Assessing and Strengthening Capacity of National Immunization Services. Background paper.

6. GAVI Alliance (2006) Health system strengthening. Geneva: GAVI Alliance Secretariat.

7. Raviglione MC, Uplekar MW (2006) WHO's new Stop TB Strategy. Lancet 367: 952-955.

8. Caines K (2005) Key evidence from major studies of selected Global Health Partnerships. London: DFID Resource Center.

9. GAVI Alliance (2007) Revised Guidelines for GAVI Alliance Health System Strengthening (HSS) applications.

10. Bennett S, Fairbank A (2003) The systemwide effects of the Global Fund to Fight AIDS, Tuberculosis and Malaria: a conceptual framework. Bethesda Maryland: Partners for Health ReformPlus.

11. WHO (2007) The Global Fund Strategic Approach to Health Systems Strengthening. Report from WHO to The Global Fund Secretariat. Geneva: World Health Organisation.

12. World Bank (2007) Healthy Development. The World Bank Strategy for Health,

Nutrition, and Population Results Washington: The World Bank.

13. UNAIDS (2007) Policies: HIV treatment, care and support. Geneva: UNAIDS.

14. Buvé A, Kalibala S, McIntyre J (2003) Stronger health systems for more effective HIV/AIDS prevention and care. Int J Health Plann Manage 18 Suppl 1: S41-51.

15. Anonymous (2007) The Global Fund expands its role. Bull World Health Organ 85: 742-743.

16. Office of the US Global AIDS Coordinator (2004) The President's Emergency Plan for AIDS Relief. U.S. Five-year global HIV/AIDS strategy.

17. PEPFAR (2006) Report on Work Force Capacity and HIV/AIDS.

18. Committee for the Evaluation of the President's Emergency Plan for AIDS Relief (PEPFAR) Implementation (2007) PEPFAR Implementation: Progress and Promise. Executive Summary. Washington: Institute of Medicine of the National Academies.

19. USAID (2007) Our work: a better future for all. Washington: U.S. Agency for International Development.

20. Mahendradhata Y, Lambert ML, Van Deun A, Matthys F, Boelaert M, et al. (2003) Strong general health care systems: a prerequisite to reach global tuberculosis control targets. Int J Health Plann Manage 18 Suppl 1: S53-65.

21. Global Health Council (2003) Reducing Malaria’s Burden Evidence of Effectiveness for Decision Makers. Technical Report. Washington: Global Health Council.

22. WHO (2007) Global Plan to Combat Neglected Tropical Diseases 2008-2015. Geneva.

23. APOC (2007) Redynamisation des soins de santé en Afrique sub-saharienne. Renforcement potentiel des systèmes de santé par le traitement sous directives communautaires. Geneva: World Health Organisation.

24. Mills A (2007) Strengthening health systems. The Commonwealth Health Ministers Book 2007.

1. See also the GFATMT’s site [www.theglobalfund.org/en/links_resources/library/evaluation_ framework/1/](http://www.theglobalfund.org/en/links_resources/library/evaluation_ framework/1/)). [↑](#footnote-ref-2)
2. See also http://www.aidstreatmentaccess.org/mtt3pepfar.htm [↑](#footnote-ref-3)
