# Supplementary material for: Global Health Actors Claim To Support Health System Strengthening—Is This Reality or Rhetoric?
Source: PLoS Med. 2009 Apr 28;6(4):e1000059. doi: 10.1371/journal.pmed.1000059 (PMC2667637; doi:10.1371/journal.pmed.1000059)
Supplement: Text S2 — A summary of policies and interventions of global health actors in the domain of health system strengthening. (60 KB DOC) [file pmed.1000059.s002.doc]

### Supporting information file 1

# Methodology

We carried out a desk study on the interventions of global health actors (GHA) in the domain of health system strengthening.

The terms ‘global health initiative’ or ‘global health partnership’ are much used but ill defined. They cover very different groups of actors [1]. In this paper, we discuss the international initiatives and key agencies working in the domain of health system strengthening. Acknowledging their different nature, we use in this paper the term global health actor (GHA) for reasons of simplicity.

The main thrust was a non-systematic review of the published and grey literature with the aim to uncover key examples of health system strengthening definitions and interventions. Additional fine-tuning occurred as a result of a presentation at the Geneva Health Forum 2008 and during discussions with expert colleagues.

Document retrieval: The literature search was structured in two main phases. First, we searched the websites of GHA for their key strategic documents and for descriptions of their health system strengthening interventions. This resulted in policy documents, intervention reports and assessments. In a second phase, we searched for published academic papers by using the following key words (individually and in combination) in the PubMed and Google Scholar search engines: health system, global health initiative, health system strengthening, international aid and vertical programme, over the period 1996-2008. This resulted in over 1000 hits for each search. AC and BM selected promising papers on the basis of scanning of titles and abstracts. We used the snowballing approach to find additional relevant academic papers and grey literature. All in all, this resulted in a selection of 99 academic papers and 23 grey publications.

The text analysis: authors BM and AC both read all documents and searched for (1) definitions of ‘health system’ and ‘health system strengthening’, (2) interventions that explicitly aim at strengthening health systems, (3) assessments of effectiveness of HSS, (4) specific assessments of negative effects. These authors compared notes and summarised the findings. The analytical approach consisted of concept mapping, i.e. identifying the constructs of our key issues as presented in the retrieved documents [2]. It should be noted that we did not go into statistical analysis. The three authors then compared the constructs and designed a typology, which was used to present the findings.

For the assessment of effectiveness and especially the negative effects, we additionally scanned the academic and grey literature for analytical frameworks. This resulted in three papers [3], [4], [5], which we found interesting and complementary rather than sufficient in se. We therefore designed an alternative framework, which we did not present in the current manuscript due to length limits. On the basis of this framework, we compared the constructs of HSS interventions with the actual implementation on the ground.

Limitations of our review include the difficulty of assessing the level of saturation: given that we searched for a multi-layered subject, interesting information could be contained in reports not indexed by our key words.

### References

1. Caines K (2005) Key evidence from major studies of selected Global Health Partnerships. London: DFID Resource Center.

2. Trochim W, Kane M (2005) Concept mapping: an introduction to structured conceptualization in health care. Int J Qual Health Care 17: 187-191.

3. Travis P, Bennett S, Haines A, Pang T, Bhutta Z, et al. (2004) Overcoming health-systems constraints to achieve the Millennium Development Goals. Lancet 364: 900-906.

4. Buse K, Harmer AM (2007) Seven habits of highly effective global public-private health partnerships: practice and potential. Soc Sci Med 64: 259-271.

5. McKinsey & Company (2005) Global health partnerships: assessing country consequences. Bill & Melinda Gates Foundation.
